# Safety and Technical Success of Percutaneous Left Main Coronary Artery Stenting

**DOI:** 10.12669/pjms.304.4859

**Published:** 2014

**Authors:** Liaqat Ali, Shahid Nawaz Malik, Abdullah Bin Khalid, Mehboob Sultan, Nadeem Sadiq

**Affiliations:** 1 Liaqat Ali, FCPS (Medicine), Department of Cardiology, Pakistan Institute of Medical Sciences, Islamabad, Pakistan.; 2 Shahid Nawaz Malik, MRCP, Interventional cardiologist, Head of Department of Cardiology, Department of Cardiology, Pakistan Institute of Medical Sciences, Islamabad, Pakistan.; 3 Abdullah Bin Khalid, Dow University of Health Sciences, Karachi, Pakistan.; 4 Mehboob Sultan, FCPS (Pediatrics), AFIC/NIHD. Rawalpindi, Pakistan. Armed Forces Institute of Cardiology & National Institute of Heart Diseases AFIC/NIHD, Rawalpindi, Pakistan.; 5 Nadeem Sadiq, FCPS (Pediatrics), Armed Forces Institute of Cardiology & National Institute of Heart Diseases AFIC/NIHD, Rawalpindi, Pakistan.

**Keywords:** Left main coronary artery, Coronary artery bypass graft

## Abstract

***Objective:*** Critical stenosis of left main coronary artery (LMCA) has always remained a challenge for interventional cardiologists. Conventionally Coronary Artery Bypass Grafting (CABG) is done for these patients but recently Percutaneous Coronary Intervention (PCI) is also being tried more frequently**, **but data of PCI is scarce in this regard. Our objective was to determine the safety and technical success rate of percutaneous left main coronary artery stenting.

***Methods: ***This was 12 month follow up study conducted at Pakistan Institute of Medical Sciences (PIMS), Islamabad from 11^th^ Jan 2012 to 11^th^ Jan 2013. All symptomatic patients who underwent coronary angiogram at PIMS and were found to have either isolated LMCA disease or coexisting osteal Left Anterior Descending (LAD) artery disease were potentially eligible for the study. Patients who had previous surgical treatment for coronary artery disease and those with renal dysfunction requiring dialysis were excluded. Patients were counselled in detail regarding the pros and cons of PCI versus CABG.Those who opted for PCI were included in the study. All these patients were treated with percutaneous left main coronary artery stenting with or without osteal LAD stenting.

***Results: ***Seventy two patients had LMCA disease during angiogram. Fifteen patients opted for CABG. Four patients did not meet the inclusion criteria, whereas 53 patients were finally enrolled**. **Mean age of patients were 55.45±10.275 years. Twenty nine patients were with acute coronary syndrome and 22 presented with unstable angina.PCI with stenting was technically successful in all patients. One patient died three months after PCI, there was no other mortality.

***Conclusion: ***Our study showed that Percutaneous Coronary Intervention (PCI) to LMS has good technical success rate; the safety of the procedure is also acceptable.

## INTRODUCTION

Significant left main coronary artery (LMCA) disease has been found in 3% to 5% of all patients who undergo coronary angiography and in 10% to 30% of patients who undergo bypass surgery.^1^^-^^4^ Critical LMCA stenosis puts patients at high risk of cardiovascular events because of the extent of jeopardized myocardium and associated multi-vessel coronary artery disease and, therefore, it has been considered as the most important coronary lesion in terms of prognosis. Current practice guidelines suggest coronary artery bypass grafting (CABG) as the standard procedure for patients with unprotected LMCA disease^5^^-^^7^ primarily because long-term outcomes of surgical revascularization are superior to those of medical treatment.^8^^-^^10^ However, because of anatomic accessibility and other characteristics, percutaneous coronary intervention (PCI) for LMCA disease was attractive to the interventional cardiologist, and data from several archives showed its feasibility and short and midterm effectiveness. Nevertheless, PCI for LMCA disease has been limited to surgically high-risk patients and those with protected LMCA disease, or has been used as bailout procedures in patients with angioplasty complications.

Nonetheless, current improvements in interventional techniques and adjunctive pharmacology have challenged the orthodox wisdom that significant LMCA stenosis^11^^-^^13^ should be cured surgically. The introduction of coronary stenting has led to a reassessment of the role of PCI as a practical treatment option for LMCA disease,^14^^-^^17^ and the widespread availability of drug-eluting stents (DES), together with enhanced stenting techniques, has lowered the threshold for use of PCI, instead of CABG, in patients with LMCA disease.^18^ The clinical experience with PCI for LMCA disease involves a wide spectrum of clinical and angiographic subcategories of such patients. However, there has been little evaluation of the long-term safety and efficiency of PCI with stenting for LMCA disease, and no randomized trial has compared the two primary interventions (PCI versus CABG) in a large population.^19^ We have therefore reviewed recent advances and the current status of percutaneous versus surgical treatment for LMCA disease, focusing on whether PCI is an alternative to or a possible replacement for CABG in these patients.^20^

The rationale of the study was to recognize the success rate of percutaneous left main coronary artery along with determination of safety till 12 months of follow-up.

## METHODS

***Inclusion / Exclusion Criteria: ***All patients with age less than 70 years and having a history of coronary heart disease were included in the study. Whereas all patients who had previous surgical treatment for coronary artery disease, extreme left-dominant coronary artery perfusion, significant carotid stenosis requiring treatment and renal dysfunction requiring dialysis were excluded.

***Data Collection Procedure: ***All patients who have history of coronary artery disease or who presented with acute coronary syndrome and were found to have either isolated LMCA disease or Osteal LAD disease along with LMCA were potentially eligible for enrollment. Patients were counselled in detail regarding pros and cons of PCI versus CABG.High risk informed consent was taken from those who opted for PCI and were subsequently enrolled in the study. All procedures were performed by a single operator who has extensive experience of interventional cardiology Angiogram was performed in Cardiac Catheterization laboratory at PIMS. All these patients were treated with PCI along with DES. In all patients everolimus drug eluting stents were used.Post stenting all patients were kept in Coronary Care unit (CCU) where their hemodynamics along with continuous ECG monitoring was done. All patients were started on dual antiplatelet therapy namely Aspirin and Clopidogrel. Those patients who had history of acute coronary syndrome were also treated with the antiplatelet agent Tirofiban. If patients remained stable for 24 hours they were moved to cardiology ward and discharged later on.All patients were followed up in cardiology outpatient Department fortnightly for the first two months and then monthly for the next 12 months.

***Statistical analysis: ***Data was recorded on predesigned proforma and analyzed on SPSS version 17.0. Mean and standard deviation was calculated for quantitative variables whereas frequency and percentages were calculated for qualitative variables.

## RESULTS

**Fig.1:**
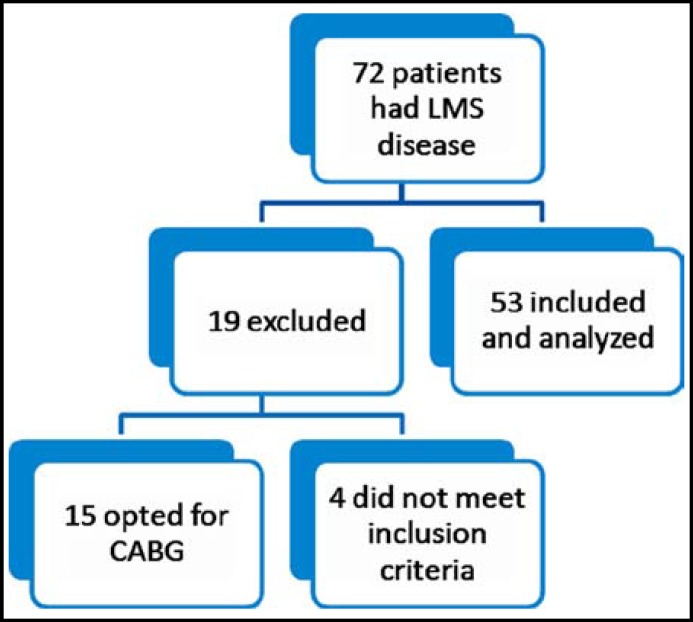
Study flow chart

Seventy two patients had LMS disease during angiogram, out of which 15 patients opted for CABG, 4 patients did not meet the inclusion criteria and thus 53 patients were finally enrolled (Fig.1). Patients were aged 35 – 68 years with mean age of 55.45+10.275 respectively. Male patients were 40 (75.47%) and female patients were 13 (24.52%) in number. Twenty nine (52.8%) patients presented with acute coronary syndrome and 22 (41.5%) patients presented with unstable angina (Table-I). On coronary angiograms 45(84.9%) patients had LMCA stenosis along with Osteal LAD stenosis while the rest had isolated LMCA disease, out of these 45 patients 22 (41.5%) had ostial left main coronary artery disease, 13 (24.5%) had distal and 10 (18.8%) had mid LMS. Out of the 5 patients who developed Ventricular Tachycardia during PCI were successfully reverted to sinus rhythm by cardioversion. PCI with stenting was technically successful in all patients. On follow up at one month none of the patients had any symptoms of coronary artery disease. Their ECG did not show any new change. At 3^rd^ month one of the patient who was a 45 year old male who had undergone a coronary stenting three months back due to an acute coronary syndrome (STEMI) presented to the Emergency department with acute chest pain. His ECG showed ST elevation in septal leads with a positive AVR, while his bedside echo in the emergency department showed normal LV dimensions with akinetic apical and mid septum, and hypokinetic apical anterior wall with an LVEF of 40-45%. Cath lab team was informed for an emergency procedure and preparation of the patient for an emergency diagnostic and therapeutic PCI was started. In the meantime he suddenly developed cardiac arrhythmias, seen as runs of VT/VF on the cardiac monitor. DC shocks were given as per ACLS guidelines but the patient could not be reverted and therefore died soon thereafter. There was no other mortality at 12 months (Table-II).

## DISCUSSION

Significant LMCA disease is a high-risk lesion that compromises blood flow to approximately 75% of the heart. Its prevalence in patients undergoing coronary angiography is 2.5% to 10%, and typically it coexists with other significant narrowing of the coronary tree. Medical treatment of LM disease has unacceptably high mortality rates.^7^^,^^21^ Early observational studies demonstrated that long-term prognoses of patients with medically treated LMCA disease were poor, with 3-year survival rates of 50%.^11^

**Table-I:** Demographics of the study subjects

***Age (Years)***	***55.45*** ***+*** ***10.275***
Male/Female	40/13
Acute coronary syndrome	29(52.8%)
Unstable Angina	22(41.5%)
Stable angina	2(3.7%)

**Table-II:** Procedure findings

***VARIABLES***	***N(%)***
Isolated LMCA disease	8(15%)
Osteal LAD involvement	45(84%)
Ventricular Tachycardia during procedure	5(9.4%)
Death	1(1.8%)
Technical success	53(100%)

Traditionally the main mode of treatment for LMCA has been CABG with PCI being reserved for surgically poor candidates. But with the advent of improvement in techniques and drug eluting metallic stents, new interest in treating LMS stenosis with PCI has emerged.

In our study, the patients affected with LMCA which were later treated with PCI had mean ageof 55.45+10.275.Whereasin other studies majority of patients presented at an advanced age.^23^ This highlights the fact that not only is the disease burden onthe Indian subcontinent estimated to be the highest worldwide but there also is a markedly earlier progression of disease within the resident population.^22^

In our study the frequency and percentage of patients suffered from unstable angina were 22 (43.1%) with all normal base line investigations. Similarly, the percentage of unstable angina was 46 in the study conducted by Lee et al.^23^

In our study we had one mortality at 3 months which is in line with other studies done in this regard.^24^ In a comparable study conducted in 2013 by Ng W et al showed 11 cardiac deaths (1 in-hospital and the remainder beyond 30 days).^4^ In our study we do not have any significant adverse effects on follow-up of our patients, other studies also reported a very low incidence of adverse effects post PCI.^24^

The limitation of this study is that we do not have a long term follow up beyond 12 months to document long term safety. Secondly this was not a randomized controlled trial to compare the two techniques namely PCI versus CABG. Nevertheless this is one of the initial studies of its kind from this region clearly showing good technical success as well as short term safety of PCI in LMS.

## CONCLUSION

Our study showed that PCI to LMCA has good technical success rate; the short term safety of the procedure is also acceptable. The result suggests potential need for a large, multicenter, randomized study with long-term follow up to provide a basis for re-evaluation of treatment guidelines for the treatment of left main coronary artery disease.
